# Symptom profile and short term outcome of catatonia: an exploratory clinical study

**DOI:** 10.1186/s12888-015-0554-2

**Published:** 2015-07-22

**Authors:** Benyam Worku, Abebaw Fekadu

**Affiliations:** Department of Psychiatry, College of Health Sciences, School of Medicine, Addis Ababa University, PO Box 9086, Addis Ababa, Ethiopia; Department of Psychological Medicine, Centre for Affective Disorders, Institute of Psychiatry, King’s College London, London, UK

**Keywords:** Catatonia, Catatonia syndrome, Low and middle income country, Ethiopia

## Abstract

**Background:**

Catatonia is a potentially life-threatening but treatable neuropsychiatric condition. Although considered more common in low income countries, data is particularly sparse in these settings. In this study we explore the symptomatology, treatment, and short-term outcome of catatonia in Ethiopia, a low income country.

**Methods:**

The study was a prospective evaluation of patients admitted with a DSM-IV diagnosis of catatonia. Diagnosis of Catatonia and its severity were further assessed with the Bush-Francis Catatonia Rating Scale (BFCRS).

**Results:**

Twenty participants, 5 male and 15 female, were included in the study: 15 patients (75 %) had underlying mood disorders, 4 patients (20 %) had schizophrenia and 1 patient (5 %) had general medical condition. The most common catatonic symptoms, occurring in over two-thirds of participants, were mutism, negativism, staring and immobility (stupor). Eighteen (90 %) of the twenty patients were on multiple medications. Antipsychotics were the most commonly prescribed medications. ECT was required in seven patients (35.0 %). Dehydration, requiring IV rehydration, and infections were the most important complications ascribed to the catatonia. These occurred in seven patients (25 %). Almost all patients (n = 19/20) were discharged with significant improvement.

**Conclusion:**

This study supports the growing consensus that catatonia is most often associated with mood disorders. Overall prognosis appears very good although the occurrence of life-threatening complications underlines the serious nature of catatonia. This has implication for “task-shifted” service scale up plans, which aim to improve treatment coverage by training non-specialist health workers to provide mental health care in low income countries. Further larger scale studies are required to clarify the nature and management, as well as, service requirements for catatonia.

## Background

Catatonia is a neuropsychiatric syndrome characterized by motor, cognitive, affective, and sometimes autonomic disturbances. More than 40 signs of catatonia have been described in the literature [1–3] although stupor, mutism, negativism, and posturing are some of the most commonly recognized signs [4, 5]. Diverse causes of catatonia, ranging from primary psychiatric disorders to idiopathic causes have been described [6]. In addition to the interest in the behavioural symptoms, neurobiological basis, epidemiology and management, [7–10], several studies have reported that anxiety is the most common subjective feeling reported by catatonic patients upon recovery [7–9, 11–13].

Since Karl Kahlbaum’s original description in his historical monograph entitled *catatonia or tension insanity* [14, 15] over a century ago, catatonia has been a subject of constant fascination and controversy. While some consider it to be a potentially distinct syndrome deserving its own nosological status [3, 16, 17], others are unsure [18, 19]. Even though catatonia has been strongly associated with schizophrenia due to the enduring influence of Emil Kraeplin and Eugene Bleuler [20], many patients with catatonia have mood disorders, while a sizeable proportion of patients with schizophrenia have catatonic features.[3, 13, 21–25]. Related to the historical views is the assumption that that there is a decline in the prevalence of catatonia in high-income countries [26, 27], while it remains relatively common in low income countries [3, 28]. For example, the International Pilot Study of Schizophrenia, a cross-country cohort study of schizophrenia involving both high and low income countries, has described catatonia to be more common in low income countries [29]. However, systematic studies on the subject are limited, particularly in low income countries. The possible contribution of genetics, socio-cultural, environmental and public health factors as well as change in clinical practice to the difference in prevalence of catatonia between the high income and low income countries is not settled [29, 30]. There are also uncertainties around the best treatment practices even if current treatment recommendations favor the use of benzodiazepines with electro-convulsive therapy (ECT) reserved for patients who fail to respond to adequate treatment with more conservative approaches [18]. For example, when catatonic symptoms occur in the context of chronic schizophrenia, response to treatment with benzodiazepines has been less favorable [31, 32]. The role of antipsychotics is still controversial. The common recommendation is to avoid antipsychotics at least during the early phases of treatment to avoid antipsychotic-associated neuroleptic malignant syndrome (NMS), which has been believed to occur in up to 10 % of patients with catatonia treated with antipsychotics [33–35]. The prognosis of catatonia is variable, ranging from good recovery to acutely rapid progression to death (often complicated by renal failure). Instituting appropriate treatment promptly may prevent complications and improves outcome [36–38]. Despite the recent revival of interest in the study of catatonia [16], contributions to the field from low income countries has been scarce and virtually non-existent in sub-Saharan Africa. The primary purpose of this study was to describe the profile of symptoms occurring in catatonia, and to evaluate the treatment and outcome of catatonia in a low income country. It was also hoped that the study would help to clarify effective management choices in low income countries.

## Methods

### Study design and setting

The study design was a facility based prospective observational study and was conducted at Amanuel hospital. Amanuel is the only psychiatric hospital in Ethiopia, although psychiatric care is provided in all the main cities of the country as part of integrated care provision. Patients from across the country use Amanuel hospital, but as would be expected, most users come from Addis Ababa or the surrounding areas [39]. The hospital has 280 beds and many of the psychiatrists of the country work in the hospital. The hospital service is organized in case teams based on the main diagnostic categories. The treatments available for psychosis are primarily first generation antipsychotics, with two second generation antipsychotics (olanzapine and resperidone), which are not always available. The main benzodiazepine consistently available is diazepam. Lorazepam is not available in the hospital or the country. The hospital runs the only electro-convulsive therapy (ECT) service in the country. Admitted patients, particularly those with more severe illness, are required to have their families with them.

### Participants

The study participants were recruited between June and September 2011 from the inpatient services of Amanuel hospital. Cases confirmed to have catatonia syndrome by the treating psychiatrists according to the Diagnostic and Statistical Manual of Mental Disorders, 4^th^ revision (DSM-IV) [4] were referred to the research team for further assessment. Treatment was initiated and managed by the treating psychiatrist.

#### Inclusion criteria

Patients, age 15 years and above, admitted to the hospital with a diagnosis of catatonia syndrome during the study period and having two or more items positive from the BFCRS screening criteria were recruited into the study.

#### Sample size

The study was exploratory and hypothesis generating. Therefore, cases admitted to the hospital consecutively during the study period constituted the study sample and no formal sample size estimation was carried out.

### Measures

#### Baseline assessment

A simple historical, clinical and socio-demographic inventory developed for this study and BFCRS were administered at baseline. The inventory allowed extraction of information from clinical records and recording of additional information obtained from direct interview. Information of interest included clinical presentation at the index admission, past psychiatric history and family history of similar illness. Presence and severity of catatonia was assessed at baseline using the BFCRS. The BFCRS has 23 items and is a widely used scale [40] with established reliability and validity [1]. The BFCRS serves both to screen for catatonia and to estimate its severity. The first 14 items of the scale serve as screening tool. Positive response to two of these screening items confirms the occurrence of catatonia. These 14 items and a set of nine additional items are used to rate for the severity of the catatonia.

#### Follow-up assessment

The BFCRS was administered at discharge. All treatments (including doses of drugs) were recorded using a separate treatment recording form. A semi-structured questionnaire was also administered to five fully recovered patients to explore retrospectively subjective feelings during the catatonic state.

#### Raters

A senior resident administered all the questionnaires.

### Data analysis

Data were entered into version 20 of the Statistical Packages for Social Sciences (SPSS). Data analysis was also carried out in SPSS. Analyses focused on description of baseline demographic and clinical profiles, including the profile of catatonic symptoms. Simple comparative analyses were conducted focused on comparing the prevalence of the different catatonic symptoms in patients with underlying diagnosis of schizophrenia and mood disorders. Outcome data obtained prospectively was also handled through simple descriptive analytic methods.

The study was approved by the Scientific Committee of the Department of Psychiatry, Addis Ababa University, and the Ethics Committee of Amanuel hospital. Consent process relied on obtaining written permission from guardians of patients who were accompanying patients in the wards. Permission was obtained from guardians after adequate information was provided by the study team. All seriously ill inpatients are required to be accompanied by their relatives while they are in hospital.

## Results

### Baseline demographic and clinical characteristics

A total of 20 participants were recruited during the study period. Fifteen participants were admitted for their first episode of illness, and 17 were treatment naïve and presented to seek care for the first time. The mean age (SD) of participants was 24.0 (7.8) years, ranging from 15 to 45. Most of the participants (n = 15; 75 %) were females and from rural parts of the country (n = 12; 60 %). Seventeen (85 %) were single and either students or unemployed prior to the onset of their illness (Details are provided in Table 1). Fifteen participants (75 %) had a diagnosis of mood disorder with catatonic features; all of these had depressive disorder except one who was admitted with a depressive phase of bipolar I disorder. Four participants (20 %) had a diagnosis of catatonic schizophrenia and one participant (5 %) a diagnosis of catatonia due to general medical condition (GMC), the GMC being Human Immunodeficiency Virus infection/Acquired Immunodeficiency Syndrome (HIV/AIDS). Participants with schizophrenia were older, with a mean age (SD) of 29.8 (10.5) years compared to those with a diagnosis of underlying mood disorders (Mean (SD) =23.6 (6.6) years. Comorbid substance use was elicited only in one patient who was admitted with a diagnosis of schizophrenia. Further details are presented in Table 1.

**Table 1:** Demographic and clinical characteristics of participants at baseline

Characteristics		Number	Percent
Demographic characteristics			
Sex			
	Male	5	25
	Female	15	75
Age			
	15-24	12	60
	25-34	5	25
	35-49	3	15
Employment before onset of illness			
	Unemployed	5	25
	Student	9	45
	Farmer	2	10
	Housewife	3	15
	Undetermined	1	5
Educational background			
	Non-literate	8	40
	Literate	12	60
Marital status			
	Single	17	80
	Married	2	15
	Divorced	1	5
Clinical Characteristics			
Diagnosis			
	Schizophrenia	4	20
	Mood Disorders	15	75
	Catatonia due to General Medical Condition	1	5
Precipitating factors identified	--	7	35
Substance use history	--	1	5
Past history of catatonia	--	2	10
First service contact	--	17	85
		Mean	SD
Duration of catatonic symptoms before admission in days--Mean, SD			
	Schizophrenia	295	326
	Mood Disorders	38.6	37.3
	GMC	21	--

**Table 2:** Treatment and outcome characteristics of patients with catatonia syndrome

		Underlying diagnostic group						
		Schizophrenia		Mood Disorders		General Medical Condition		
Treatment		No	Percent	No	Percent	No.	Percent	Total (%)
	Benzodiazepines	1	25.0	12	80.0	1	100.0	14 (70)
	Antidepressants	0	0.0	9	60.0	0	0.0	9 (45)
	Antipsychotics	4	100.0	13	86.7	1	100.0	18 (90)
	Electroconvulsive therapy	2	50.0	3	20.0	1	100.0	6 (30)
Outcomes		mean	SD	mean	SD	mean	SD	Overall mean (SD)
	Length of hospital stay	40.8	15.9	33.5	30.3	39	--	34.5 (26.3)
	Bush-Francis Severity scores	3.5	2.4	2.8	6.9	0	--	2.7 (5.9)

### Catatonic symptoms

**Fig. 1:**
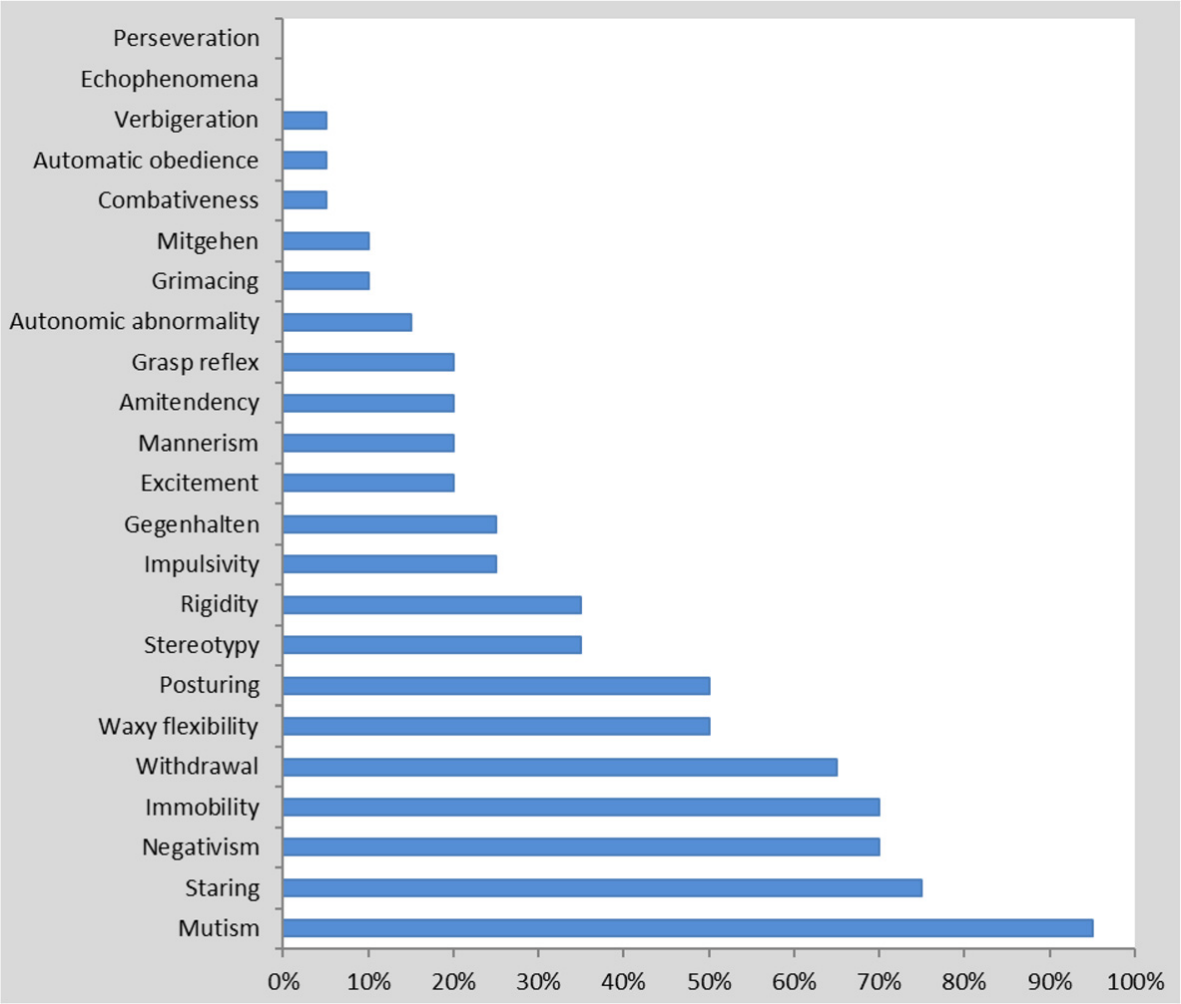
Proportion of patients with specific catatonic features from the full scale of the Bush-Francis scale

**Fig. 2:**
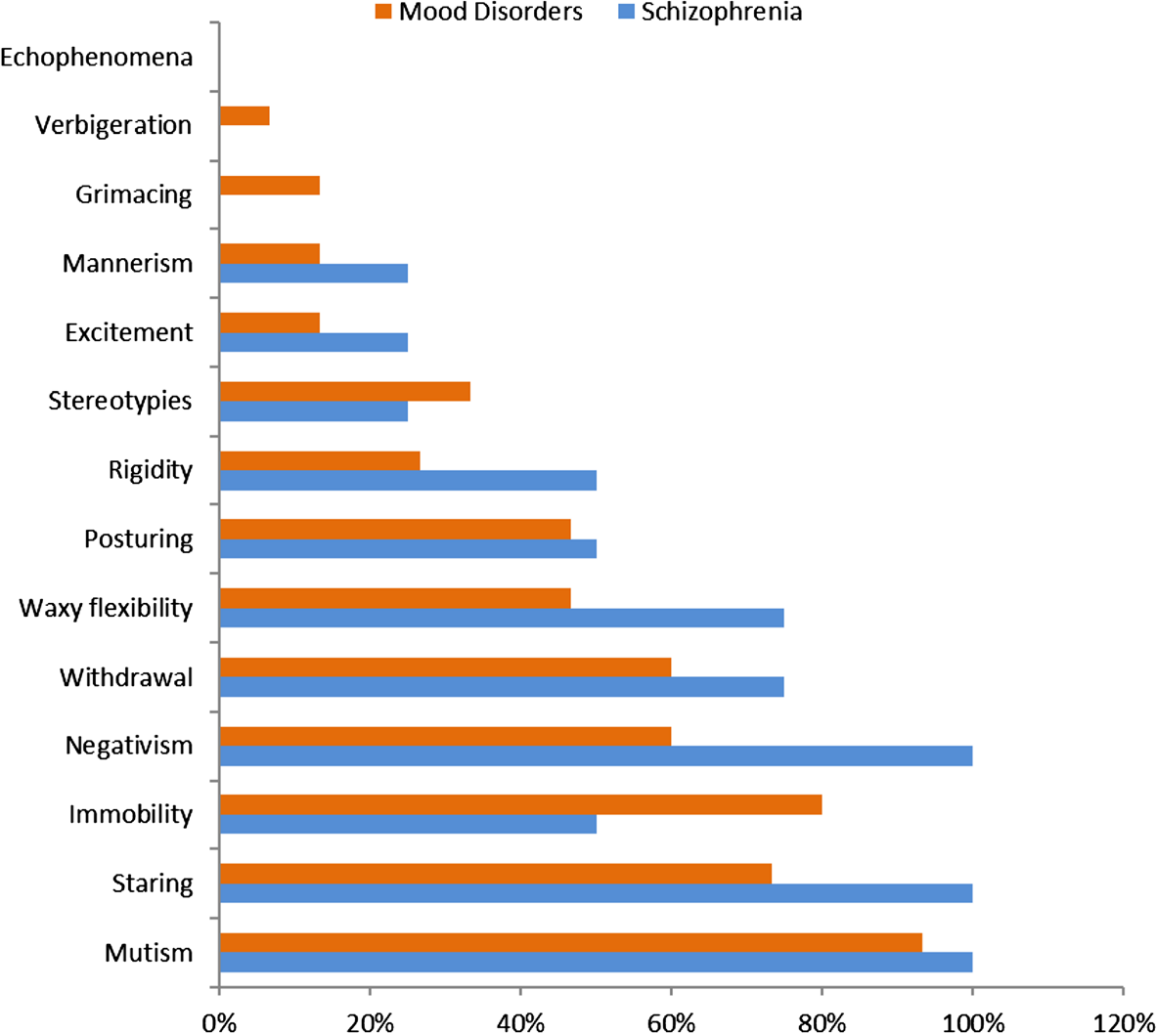
Catatonic features from the 14 screening items of the Bush-Francis scale comparing those with underlying diagnosis of schizophrenia and mood disorders

At admission, the mean (SD) BFCRS score for the whole sample was 15.9 (5.8). The participants admitted with GMC had the highest score (26.0). The mean (SD) score for those with schizophrenia was 19 (6.1) while for those with mood disorders was 14.3 (5.0). Figure 1 presents the symptom profile of the 20 participants based on the 23 items of the BFCRS. All items were rated except two, echophenomena and perseveration. However, only seven of the 23 items were found in 50 % or more of the participants: mutism (95 %), staring (75 %), negativism (70 %), immobility (stupor) (70 %), withdrawal (65 %), waxy flexibility (50 %) and posturing (50 %). In addition to the items that were completely absent (echophenomena and perseveration), verbigeration, automatic obedience and comabtiveness were the lowest rated occurring in only 5 % of the patients. Exploratory comparison focusing on schizophrenia and mood disorders showed that participants with schizophrenia have more catatonic symptoms (Figure 2). The main exception is immobility (stupor), which was higher among those with mood disorders. Slight increase was also observed in stereotyped behavior, grimacing and verbigeration among participants with mood disorders. All participants with schizophrenia (n = 4) had mutism, staring and negativisim. The patient with catatonia due to GMC had 10 of the 23 symptoms (excitement, mutism, posturing, stereotypy, mannerism, rigidity, negativism, withdrawal, impulsivity and gegenhalten). The mean (SD) duration of catatonic symptoms among patients with schizophrenia was 295 (326) days while the comparable figures for those with mood disorders were 38.6 (37.3) days. The patient with GMC had the symptoms for 21 days prior to admission.

Overall, possible factors that were considered relevant triggers for the catatonia were elicited in five patients with major depressive disorder and the patient with GMC. In those with major depressive disorders, three patients had academic problems while two were returnees from the Middle East.

### Treatment and outcomes

Four classes of treatment were used (Table 2). Antipsychotics were the most commonly used (n = 19/20; 95 %) followed by benzodiazepines (n = 15/20; 70 %) and antidepressants (n = 9; 60 %). ECT was also used in seven cases (35 %). Where benzodiazepines were used, they were initiated before the administration of antipsychotic medications. Among the antipsychotics, olanzapine was used in 11/19 cases in doses ranging from 5 mg to 15 mg. Haloperidol was used in seven cases in doses ranging from 1.5 mg to 5 mg. Chlorpromazine (200 mg) was also used in one case with mood disorder. Electroconvulsive therapy was used for three cases with schizophrenia, three cases with mood disorders and in the only case of GMC. Diazepam in doses of 5–20 mg, bromazepam (3–6 mg) and clonazepam (0.5 mg) were the benzodiazepines used. Amitriptyline and imipramine were used in two cases each for patients with mood disorders in doses ranging from 25 mg to 75 mg. Fluoxetine was used in the remaining five cases in doses of 20–40 mg. Eighteen of the 20 patients were treated with two or more medications from different groups or ECT.

Seven participants had complications during their hospitalization. Five of the cases had dehydration necessitating intravenous resuscitation. One case had hypostatic pneumonia and another urinary tract infection. None of the patients developed symptoms suggestive of neuroleptic malignant syndrome.

All participants were discharged with good improvement except one case that was discharged against medical advice as the family decided to take her home (Table 2). The mean (SD) discharge score of BFCRS for those with schizophrenia was 3.8 (1.9). The comparable mean (SD) score for those with mood disorders was 0.9 (1.7). While only one case with schizophrenia had a score of zero, 10 of those with mood disorders scored zero at discharge. ECT was particularly effective with five of the seven cases treated with ECT scoring zero on the BFCRS. The overall mean (SD) duration of hospital stay was 34.5 (26.3), longest for schizophrenia (mean (SD) =40.7 (15.8) days) and shortest for the cases with mood disorders (mean (SD) =33.5 (30.3) days). Patients who received benzodiazepines had the shortest duration of hospitalization while those treated with ECT had the longest.

Two patients who were diagnosed with Major Depressive Disorder (MDD) had recurrent catatonia. One of the patients with recurrent catatonia was admitted 3 years ago with a diagnosis of MDD with catatonic features that resolved after treatment with ECT. The second patient had four episodes of probable catatonia over a period of 13 years. Each episode lasted 10–15 days on average and they resolved without any treatment. This patient responded to treatment with benzodiazepines while a full course of ECT was necessitated in the first case.

It was possible to obtain subjective experience from four participants with underlying depressive disorder and one patient admitted with a diagnosis of catatonia due to GMC. All reported feeling anxious and the four patients who had immobility did not recall being unable to move their body.

## Discussion

This is the first report of catatonia syndrome from Ethiopia and one of the very few from any low income country. This study supports the existing literature that: (1) catatonia may be most commonly associated with mood disorders; (2) catatonia responds relatively well to treatment; (3) catatonia could threaten life considering the relatively high number of cases with serious complications. Given the relative paucity of systematic data on catatonia, our study may be an important addition to the literature.

### Comparison with the broader literature

The results of our study for the most part concur well with results of studies reported in the wider literature [16, 17, 19, 41]. A study summarizing data from over 1000 patients indicated that mood disorders were common causes of catatonia [3, 42]. The trend of the proportions of underlying conditions (mood disorder > schizophrenia > GMC) in our sample is consistent with other studies [20, 43, 44]. However, unlike our study where only one participant with mood disorder had bipolar disorder, many catatonic patients in some of these older studies had bipolar disorder [1, 41]. This discrepancy may partly be explainable by the fact that most of our participants with mood disorders (13 of the 15) were first episode cases. This is consistent with the fact that bipolar I disorder may often start with depression [45]. The broader demographic characteristics of our sample, specifically the mean age of participants, and the male to female ratio are consistent with other reports [24, 25, 31, 41, 46, 47].

The mean total score on the BFCRS at the initial assessment was almost equal to the score of 16.8 (SD 6.6) found by Bush and colleagues among 28 patients with catatonia [48]. The most common catatonic symptoms identified in our patients were also similar to the findings of several well designed studies [1, 13, 24, 30, 48]. The good treatment outcome observed is congruent with the conclusion of experts that prognosis is excellent provided that there is prompt detection and appropriate management. However, the very high rate of polypharmacy and lack of uniformity in management shows the need for the application of evidence based treatment protocol [36–38]. The high rate of response in patients treated with ECT is consistent with the evidence base. The longer duration of hospitalization observed in patients who ultimately required treatment with ECT is because ECT is given usually as treatment of last resort [36]. The observed higher prevalence of potentially life-threatening complications indicates the level of risk associated with catatonia and the need for robust interventions in this patient population. The current plan for scaling up mental healthcare in low and middle income countries, exemplified by the mental health gap action program proposed by the World Health Organization [49] should take into account the needs of patients with catatonia, particularly given the relatively common occurrence of catatonia and potential risks of untreated catatonia. Training of primary care workers on the manifestations and treatment of catatonia and ensuring benzodiazepines are available at primary care centres where mental health service integration is planned is essential. The absence of NMS in our sample may be due to the use of low doses of medications in most cases. The use of benzodiazepines and ECT might have also contributed given these two treatments have been considered as treatment options for NMS [44, 50]. The long duration of catatonia is worth of note. This is generally consistent with the service utilization pattern of patients with severe mental disorders in the country [51], which in turn is likely to be related to the limited availability of services [52]. However, because most patients were treatment naïve at entry into the study, treatment with antipsychotic medications is unlikely to explain the long duration of catatonia.

### Strengths and limitations

The main strength of our study is its novelty. There are extremely few studies on the subject in low and middle income countries despite the prevailing view that catatonia is common in these countries. Most of our participants were also first episode cases and less affected by the chornicity of the illness and prior treatment. Some of the motor manifestations, particularly in schizophrenia, may be markers of chronicity or medication side effects. Administration of a standard instrument prior to initiation of treatment and prior to discharge is also an important advantage to this study. To our knowledge there are very few prospective studies of catatonia in the world. Given this rarity of prospective studies, our study has the potential to contribute to our knowledge about catatonia. However, this is a relatively small study although this has been one of the main problems of studies on catatonia. In addition to the low prevalence of catatonia, challenges of recruiting patients with catatonia, difficulty of data collection and challenges of communication are likely reasons for small sample sizes in many studies of catatonia. Our study sample was consecutive but instruments were not systematically administered to all admitted patients. Instead, patients identified with catatonia by treating psychiatrists were referred to the research team. Therefore, patients with non-classical catatonia symptoms such as excitement could have been missed. It was difficult to obtain adequate description of the catatonic state from our participants. Some of the possible explanations include: the narrow window of opportunity to administer the questionnaire, residual catatonic symptoms or symptoms of the underlying illness and the possible cognitive effect of treatment modalities such as ECT [9, 11–13, 53].

## Conclusions

Despite a good overall outcome, the incidence of life-threatening complications in catatonia is not negligible. This risk has to be taken into account in the context of the current efforts to improve service coverage in Low and Middle Income Countries (LAMICs). The study also indicates an urgent need for developing evidence base for the treatment of catatonia in low income countries given the limitation of treatment options in these settings. Treatments known to be effective, particularly lorazepam should be made available in Ethiopia. Our study indicates that a study on catatonia is feasible. Further systematic studies characterizing the nature of catatonia, its etiology and treatment are essential.

## Abbreviations

BFCRS: Bush-Francis Catatonia Rating Scale
DSM-IV: Diagnostic and Statistical Manual of Mental Disorders, 4^th^ edition
ECT: Electro-convulsive Therapy
GMC: General Medical Condition
HIV/AIDS: Human Immunodeficiency Virus/Acquired Immunodeficiency Syndrome
LAMICs: Low and Middle Income Countries
MDD: Major Depressive Disorder
NMS: Neuroleptic Malignant Syndrome
SD: Standard Deviation
SPSS: Statistical Packages for Social Sciences
